# Primary Hepatic Solitary Fibrous Tumour: A Rare Mesenchymal Entity with Distinct Histopathologic and Molecular Features

**DOI:** 10.3390/diagnostics16091276

**Published:** 2026-04-23

**Authors:** Alexandra Gráczer, Tamás Lantos, Anita Sejben

**Affiliations:** 1 Department of Pathology, University of Szeged, 6725 Szeged, Hungary; 2 Department of Medical Physics and Informatics, University of Szeged, 6720 Szeged, Hungary

**Keywords:** liver cancer, solitary fibrous tumour

## Abstract

Solitary fibrous tumour is an uncommon, predominantly benign tumour of mesenchymal origin, developing mainly in the thoracic cavity and on the pleural surface, although it has been reported in a wide variety of extrapleural sites. Its occurrence in the liver is particularly rare. We present the case of a 57-year-old woman in whom a large mass was identified in the left lobe of the liver, demonstrating inhomogeneous contrast enhancement without significant compression of the abdominal vessels. The lesion measured 170 mm in its greatest diameter and severely destroyed the surrounding liver parenchyma. SFT is characterised by haphazardly arranged ovoid and spindle-shaped cells with numerous mildly staghorn-like vessels lined by flattened endothelium. It typically shows *NAB2–STAT6* gene rearrangement with CD34 and/or STAT6 positivity on immunohistochemistry. Since imaging methods are not specific regarding the nature of the lesion, pathological and immunohistochemical analyses are essential for establishing an accurate diagnosis and assessing differential diagnostic possibilities.

**Figure 1:**
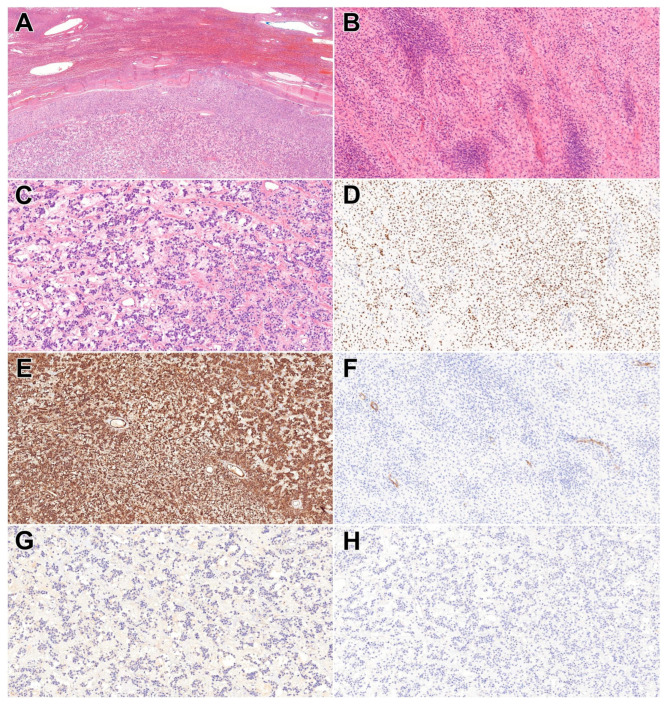
A 57-year-old woman with no significant past medical history presented for evaluation of an epigastric mass associated with nutritional compromise and unintentional weight loss. On CT, a highly vascularized tumour measuring 128 mm in its greatest diameter was identified, originating from segments II and III of the liver, displacing the stomach and the portal vein, while remaining confined to the involved hepatic lobe. Laboratory evaluation revealed tumour markers (including CEA, AFP, CA19-9, CA125, and B-hCG) within normal limits, and liver function tests were unremarkable (SGOT: 44 U/L, SGPT: 45 U/L, γ-GT: 27 U/L, ALP: 65 U/L). The nature of the lesion could not be definitively characterised based on imaging findings alone, and the lesion’s size raised suspicion of malignancy; therefore, lobectomy was performed. On gross examination, a yellowish-brown mass measuring 170 mm in greatest dimension was identified, exhibiting solid, cystic, and gelatinous components. Histologic examination revealed an encapsulated cellular proliferation ((**A**) HE, 2×) composed of haphazardly arranged ovoid and spindle-shaped cells, accompanied by numerous mildly staghorn-like vessels lined by flattened endothelium and focal cystic change ((**B**) HE, 10×). The lesion demonstrated variable cellularity ((**C**) HE, 10×). The stroma was focally edematous, with predominantly collagenous areas elsewhere. Cytologic atypia was mild, and 10 mitotic figures were identified per 10 high-power fields (HPFs). Immunohistochemical analysis demonstrated strong, diffuse nuclear expression of STAT6 in the tumour cells ((**D**) STAT6, 10×), and strong, diffuse CD34 membranous positivity ((**E**) CD34, 10×). However, the lesional cells were negative for DOG-1, excluding the possibility of a gastrointestinal stromal tumour ((**F**) DOG-1, 10×). Due to the presence of nonspecific, nest-like tumour cells at the periphery of the lesion resembling neuroendocrine neoplasms, additional immunohistochemical stains, including CKAE1/AE3 ((**G**) CKAE1/AE3—10×) and syntaxin ((**H**) Syntaxin, 10×), were performed; both yielded negative results. Based on morphological and immunohistochemical findings, the lesion was histopathologically classified as a solitary fibrous tumour (SFT). Based on the current WHO Classification, this case presents as high risk, due to the patient’s age (≥55 years), the high number of mitoses (≥4 mitoses per 10 HPFs), tumour size (>15 cm), and the eventual presence of necrosis. SFT is a fibroblastic neoplasm originating from CD34-positive dendritic interstitial cells and accounts for approximately 2% of soft tissue tumours. The lesion was first described in 1931 by Klemperer and Rabin and is characterised by ovoid to spindle-shaped cells and slightly dilated, staghorn-shaped vessels [1,2,3,4]. Previously, in the diagnosis of SFT, immunohistochemical staining for CD34, vimentin, and desmin was commonly used [5]. Later, the *NAB2–STAT6* gene fusion was identified. Because these two genes are located in proximity on chromosome 12, their rearrangement occurs over a very short genomic distance, making the fusion undetectable by conventional fluorescence in situ hybridisation (FISH) techniques [6]. This gene fusion represents the defining driver alteration in SFTs and results from an intrachromosomal inversion on chromosome 12, leading to the formation of a chimeric transcription factor. Mechanistically, the fusion converts *NAB2*, normally a transcriptional repressor, into a constitutive transcriptional activator through acquisition of the *STAT6* transactivation domain. This aberrant protein promotes oncogenesis primarily via dysregulated activation of *EGR1*-dependent transcriptional programs, thereby contributing to cellular proliferation and tumor development [7,8,9]. Based on these findings, CD34 and/or STAT6 expression by immunohistochemistry are essential [4]. Nuclear STAT6 expression is a highly sensitive (98–100%) and specific (100%) marker for SFT regardless of anatomical localisation. However, STAT6 positivity has occasionally been reported in dedifferentiated liposarcoma and deep fibrous histiocytoma, as well [10,11]. SFTs exhibit several morphological subtypes, including the fat-forming (lipomatous), giant cell–rich, and dedifferentiated variants [4]. The fat-forming variant typically arises in deeper soft tissues, whereas the giant-cell variant most frequently occurs in the periorbital region. A dedifferentiated variant is exceedingly rare and is characterised by an abrupt transition to either a low- or high-grade sarcomatous component [6]. According to the ICD-O classification, SFTs may be categorised as benign, malignant, or not otherwise specified (NOS). These categories cannot be reliably distinguished based solely on radiological findings or biopsy specimens due to limited sample size [4]. *TERT* promoter mutations correlate with the development of malignant SFT [12]. The radiological appearance of SFTs is non-specific. On contrast-enhanced imaging, it may mimic malignant lesions; therefore, accurate differentiation from other entities is essential. For example, hepatocellular carcinoma is typically negative for CD34, whereas mesothelioma characteristically demonstrates expression of CD34, vimentin, and CK7 [13,14]. Its occurrence in the liver is extremely rare and requires close clinical follow-up because of its potential for malignant transformation. According to several studies, primary hepatic SFTs do not demonstrate a predilection for any specific anatomical region of the liver, and a female predominance has been reported [11]. The malignant form tends to affect the right lobe and occurs with equal frequency in men and women [3]. According to the literature, primary hepatic SFT usually occurs in non-damaged, non-cirrhotic liver tissue, while a case has also been reported in association with polycystic liver disease [5]. Currently, no established risk factors for SFT are known. Nevertheless, a single study suggested that liver irradiation may induce the transformation of fibrous tissue into an SFT [15]. Metastatic cases most commonly involve the bones, particularly the vertebrae [16]. However, the liver may also represent a site of metastasis for SFT. For example, Zhang et al. reported a case of renal SFT with subsequent hepatic metastasis [1]. In rare cases, a paraneoplastic syndrome known as Doege–Potter syndrome may occur, presenting as therapy-resistant hypoglycemia. The symptoms are caused by tumour-produced IGF-II, which is homologous to human insulin and is primarily produced during embryonic development, reflecting the primitive mesenchymal origin of SFT. According to some studies, the presence of a paraneoplastic syndrome does not correlate specifically with malignancy [3]. Doege–Potter syndrome is most commonly associated with SFTs; however, it has also been described in mesothelioma, liposarcoma, rhabdomyosarcoma, leukaemia, lymphoma, and teratoma [17]. The currently accepted treatment of SFT is surgical resection with at least a 1 cm margin. Malignant transformation represents an infrequent event [6,12,15]. Dedifferentiated (anaplastic) SFTs exhibit progression to a high-grade sarcomatous component, which may occur with or without heterologous differentiation, such as rhabdomyosarcomatous or osteosarcomatous elements [4]. In the differential diagnostic evaluation, other primary mesenchymal hepatic neoplasms, including various sarcomas, may demonstrate spindle-cell morphology; however, these entities typically lack STAT6 expression. Inflammatory pseudotumour may clinically and radiologically resemble SFT, as it can exhibit variable delayed-phase enhancement attributable to fibrosis. Nevertheless, it is distinguished by a prominent inflammatory infiltrate and myofibroblastic proliferation, features not observed in the present case. Certain variants of hepatocellular carcinoma may also mimic SFT, primarily due to the presence of abundant fibrous stroma. However, hepatocellular carcinoma characteristically expresses hepatocellular markers such as HepPar-1, Arginase-1, and glypican-3, which are absent in SFTs. Therefore, in conjunction with histomorphological assessment, immunohistochemical analysis—particularly evaluation of STAT6 expression—is essential for establishing an accurate diagnosis [18,19,20]. Primary hepatic SFT, although rare, should be included in the differential diagnosis of large hepatic masses, particularly in the setting of normal tumour markers and preserved liver function, as well as non-specific imaging findings. Furthermore, the presence of focal neuroendocrine-like morphology in this case represents a potential diagnostic pitfall, which may result in misclassification if not corroborated by appropriate immunohistochemical studies, especially STAT6 expression. Notably, this case also exhibits features associated with an increased risk of aggressive behavior, including large tumour size and elevated mitotic activity (>4/10 HPF), underscoring the importance of meticulous histopathological evaluation and risk stratification. Given the limitations of imaging and small biopsy specimens in reliably distinguishing benign from malignant SFT, complete surgical resection followed by comprehensive pathological assessment remains essential. From a clinical management standpoint, these findings highlight the importance of prolonged follow-up, even in the absence of overtly malignant histological features, due to the potential for late recurrence or malignant transformation. We acknowledge that, as a single case report, this study has inherent limitations, particularly with respect to generalizability. The findings presented here may not be broadly applicable to all cases of SFT of the liver, given the rarity and potential heterogeneity of this entity. In addition, molecular characterization was limited to immunohistochemical analysis, and no further genetic or molecular testing (e.g., *NAB2–STAT6* fusion analysis) was performed, which could have provided additional diagnostic confirmation and biological insight. Future research should focus on larger case series or multicenter studies to better define the clinicopathological spectrum, imaging characteristics, and outcomes of hepatic SFTs. Furthermore, comprehensive molecular profiling, including next-generation sequencing and detailed analysis of *NAB2–STAT6* fusion variants, may enhance diagnostic accuracy, improve understanding of tumor behavior, and potentially identify prognostic or therapeutic biomarkers.

## Data Availability

The original contributions presented in this study are included in the article. Further inquiries can be directed to the corresponding author.

## Abbreviations

The following abbreviations are used in this manuscript:

β-hCG	Beta-human chorionic gonadotropin
γ-GT	Gamma-glutamyl transferase
AFP	Alpha-fetoprotein
ALP	Alkaline prosphatase
CA19-9	Carbohydrate antigen 19-9
CA125	Cancer antigen 125
CD34	Cluster of differentiation 34
CEA	Carcinoembryonic antigen
CK7	Cytokeratin 7
CKAE1/AE3	Cytokeratin AE1/AE3
CT	Computed tomography
DOG1	Discovered on GIST
FISH	Fluorescence in situ hybridization
HE	Hematoxylin eosin
HPF	High power field
ICD-O	International Classification of Diseases for Oncology
IGF	Insulin-like growth factor
NAB2	NGFI-A binding protein 2
NOS	Not otherwise specified
SFT	Solitary fibrous tumour
SGOT	Serum glutamic-oxaloacetic transaminase
SGPT	Serum glutamic pyruvic transaminase
STAT6	Signal transducer and activator of transcription 6
TERT	Telomerase reverse transcriptase

## References

[1] Zhang N., Zhou D., Chen K., Zhang H., Huang B. (2018). Malignant solitary fibrous tumor of the kidney with liver metastasis: A case report and literature review. J. Cancer Res. Ther..

[2] Lu J. (2024). Contrast-enhanced ultrasound imaging features of solitary fibrous tumor in the liver: A case report. Asian J. Surg..

[3] Delvecchio A., Duda L., Conticchio M., Fiore F., Lafranceschina S., Riccelli U., Cristofano A., Pascazio B., Colagrande A., Resta L. (2019). Doege-Potter syndrome by malignant solitary fibrous tumor of the liver: A case report and review of literature. World J. Gastrointest. Surg..

[4] WHO Classification of Tumours Editorial Board (2019). WHO Classification of Tumours—Soft Tissue and Bone Tumors.

[5] Korkolis D.P., Apostolaki K., Aggeli C., Plataniotis G., Gontikakis E., Volanaki D., Sebastiadou M., Dimitroulopoulos D., Xinopoulos D., Zografos G.N. (2008). Solitary fibrous tumor of the liver expressing CD34 and vimentin: A case report. World J. Gastroenterol..

[6] Tariq M.U., Din N.U., Abdul-Ghafar J., Park Y.K. (2021). The many faces of solitary fibrous tumor; diversity of histological features, differential diagnosis and role of molecular studies and surrogate markers in avoiding misdiagnosis and predicting the behavior. Diagn. Pathol..

[7] Ren C., D’Amato G., Hornicek F.J., Tao H., Duan Z. (2024). Advances in the molecular biology of the solitary fibrous tumor and potential impact on clinical applications. Cancer Metastasis Rev..

[8] Doyle L.A., Vivero M., Fletcher C.D., Mertens F., Hornick J.L. (2014). Nuclear expression of STAT6 distinguishes solitary fibrous tumor from histologic mimics. Mod. Pathol..

[9] Salguero-Aranda C., Martínez-Reguera P., Marcilla D., de Álava E., Díaz-Martín J. (2021). Evaluation of *NAB2-STAT6* fusion variants and other molecular alterations as prognostic biomarkers in a case series of 83 solitary fibrous tumors. Cancers.

[10] Cheah A.L., Billings S.D., Goldblum J.R., Carver P., Tanas M.Z., Rubin B.P. (2014). STAT6 rabbit monoclonal antibody is a robust diagnostic tool for the distinction of solitary fibrous tumour from its mimics. Pathology.

[11] Doyle L.A., Tao D., Mariño-Enríquez A. (2014). STAT6 is amplified in a subset of dedifferentiated liposarcoma. Mod. Pathol..

[12] Park H.K., Yu D.B., Sung M., Oh E., Kim M., Song J.Y., Lee M.S., Jung K., Noh K.W., An S. (2019). Molecular changes in solitary fibrous tumor progression. J. Mol. Med..

[13] England D.M., Hochholzer L., McCarthy M.J. (1989). Localized benign and malignant fibrous tumors of the pleura. A clinicopathologic review of 223 cases. Am. J. Surg. Pathol..

[14] Debs T., Kassir R., Amor I.B., Martini F., Iannelli A., Gugenheim J. (2014). Solitary fibrous tumor of the liver: Report of two cases and review of the literature. Int. J. Surg..

[15] Perini M.V., Herman P., D’Albuquerque L.A., Saad W.A. (2008). Solitary fibrous tumor of the liver: Report of a rare case and review of the literature. Int. J. Surg..

[16] Ye X., Tang X., Li F., Lin Y. (2023). A giant malignant solitary fibrous tumor in the liver: A case report. Asian J. Surg..

[17] Bodnar T.W., Acevedo M.J., Pietropaolo M. (2014). Management of non-islet-cell tumor hypoglycemia: A clinical review. J. Clin. Endocrinol. Metab..

[18] Lin H., Liu Y., Wei Y., Guan X., Yu S., Man Y., Deng D. (2024). Characteristics of imaging in hepatic inflammatory pseudotumors: A comparison between IgG4-related and IgG4-unrelated cases. Insights Imaging.

[19] Choi J.H. (2025). Inflammatory myofibroblastic tumor: An updated review. Cancers.

[20] Akbulut S., Sahin T.T. (2026). Hepatocellular carcinoma arising from ectopic liver tissue: A systematic review of the literature. Eur. J. Gastroenterol. Hepatol..

